# Long-term trends and future projections of the burden of diabetic nephropathy in China: a comprehensive analysis of GBD data from 1990 to 2036

**DOI:** 10.3389/fendo.2025.1681689

**Published:** 2025-10-08

**Authors:** Yan Zhang, Dong Hou, Zihui Chai, Xizi Li, Chuchu Shan, Yuetong Zhao, Siyuan Song, Ying Tan, Jiangyi Yu

**Affiliations:** ^1^ Department of Endocrinology, Jiangsu Province Hospital of Chinese Medicine, Affiliated Hospital of Nanjing University of Chinese Medicine, Nanjing, China; ^2^ The First Clinical Medical College, Nanjing University of Chinese Medicine, Nanjing, China; ^3^ School of Integrated Chinese and Western Medicine, Gansu University of Chinese Medicine, Lanzhou, China

**Keywords:** diabetic nephropathy (DN), Global Burden of Disease (GBD), inequality, prediction, joinpoint regression analysis, age-period-cohort analysis (APC)

## Abstract

**Background:**

Diabetic nephropathy (DN) is a prevalent and serious microvascular complication of diabetes that poses a significant public health challenge and negatively impacts quality of life in China. The objective of this study was to evaluate the disease burden of type 2 diabetic nephropathy in China and to predict the trend of this burden over the next 15 years.

**Methods:**

This study used the Global Burden of Disease (GBD) system to analyze trends in the disease burden of type 2 DN in China between 1990 and 2021. The study utilized prevalence, incidence, deaths, years of life lost (YLLs), years lived with disability (YLDs), and disability-adjusted life years (DALYs) for DN, along with their 95% uncertainty intervals (UIs). Secondly, joinpoint Regression, age-period-cohort, and decomposition analyses were employed to estimate the contribution of epidemiological changes to the DN burden. We used the inequality slope index (SII) and concentration index to assess absolute and relative cross-country inequalities in 1990 and 2021. Furthermore, Bayesian age-period-cohort (BAPC) models were employed to predict the future burden of DN from 2022 to 2036.

**Results:**

From 1990 to 2021, the burden of DN in China continued to increase, reaching a total of 20,911,520 cases. The age-standardized prevalence rate (ASPR) was 1,053.92 per 100,000 people. The age-standardized incidence rate (ASIR) was 16.29 per 100,000 people, and the age-standardized death rate (ASDR) was 5.64 per 100,000 people. Age-standardized disability-adjusted life years (DALYs) were 122.15 per 100,000 people. In 2021, the overall burden of DN continued to increase, with the effect of age strengthening with increasing age. The incidence rate showed a sustained upward trend. Decomposition analysis revealed that population ageing was the main cause of the increased burden of DN in China. Predictive Analysis suggests that the ASIR will continue to rise from 2022 to 2036, while the ASDR will decrease.

**Conclusion:**

DN in type 2 diabetes mellitus places a significant burden on China’s healthcare system, primarily due to an ageing population. The incidence rate is expected to increase over the next 15 years before declining. Given China’s large population and severe ageing, implementing a tiered prevention and control strategy, strengthening health education, and promoting early, effective prevention are imperative to alleviating the disease burden in China.

## 1 Introduction

As a common metabolic disorder, diabetes places a significant burden on global public health systems due to factors such as population ageing, changes in economic conditions and shifts in lifestyle habits (1). According to the latest report from the International Diabetes Federation (IDF), there are now over 460 million people with diabetes worldwide, a figure which is projected to rise to almost 800 million by 2045. In China, the estimated number of patients with diabetes in 2021 exceeded 140 million, and this figure is projected to surpass 174 million by 2045, making China the country with the largest diabetes patient population worldwide (2). Therefore, the disease burden in China remains severe.

Diabetic nephropathy (DN) is one of the most severe microvascular complications of diabetes. The primary clinical features of DN are a progressive decline in estimated glomerular filtration rate (eGFR) and/or persistent proteinuria (3). It is also closely associated with increased all-cause and cardiovascular disease-related mortality (4), resulting in a significant global disease burden. DN is the most common chronic kidney disease (CKD) and the leading cause of end-stage renal disease (ESRD), accounting for over 50% of cases worldwide (5, 6). It is estimated that 30–40% of patients with type 1 diabetes and 10–20% of patients with type 2 diabetes will ultimately develop ESRD. However, due to its higher prevalence, the majority of ESRD cases occur in patients with type 2 diabetes (7). The 2017 Global Burden of Disease Study revealed that the global prevalence of DN was 9.1%, equating to around 700 million cases and representing a 29.3% increase since 1990 (8). Independent risk factors for DN include genetics, hyperglycaemia, hypertension, dyslipidaemia, proteinuria (9), obesity, and metabolic disorders (10). Current treatments for DN include renin-angiotensin-aldosterone system (RAAS) inhibitors, novel antidiabetic drugs such as GLP-1 receptor agonists and SGLT-2 inhibitors, and mineralocorticoid receptor antagonists (MRAs), among others. Despite recent advancements in diagnostic techniques and treatment modalities, delaying the progression of DN remains challenging in clinical settings, significantly impacting patients’ quality of life and economic circumstances.

Research has shown that, in recent years, chronic kidney disease (CKD) in China has become more commonly associated with diabetes-related kidney disease. The incidence rate of this condition has increased to surpass that of chronic glomerulonephritis (11). Given China’s large population and ageing demographics, it contributes significantly to the global disease burden of diabetes-related chronic kidney disease. This results in a unique trend in the disease burden of DN in China. Therefore, analyzing the distribution characteristics of different populations and using the Global Burden of Disease (GBD) 2021 database to predict future trends can provide critical epidemiological evidence to inform the development of public health policies and enhance disease prevention awareness. This study primarily aims to describe trends in the prevalence, incidence, deaths, years lost due to disability (YLDs), years of life lost (YLLs), and disability-adjusted life years (DALYs) of DN in China, as well as to predict changes in disease burden trends over the next 15 years. Furthermore, the study provides supplementary insights in an international context through joinpoint regression analysis and cross-national inequality analysis, adding new perspectives to existing global assessments of the disease burden of DN.

## 2 Methods

### 2.1 Data sources

The data for this study were obtained from the GBD study. GBD2021 replaced all previously published estimates with estimates for the full 1990–2021 period. The analysis assessed 371 diseases and injuries and 88 risk factors using 100,983 data sources, and estimated YLDs, YLLs, and DALYs. Assessments were conducted across different age groups, genders, periods, geographic regions, and health domains (12). The core data for this paper comes from the Global Burden of Disease, Injury, and Risk Factors Study (GBD) 2021. The interactive features of the Global Health Data Exchange online query tool (https://ghdx.healthdata.org/gbd-results-tool) were mainly used to analyze the disease burden of DN in China (13). The study population included people of all ages with DN, which was then age-standardized. According to the 2021 GBD Study, data on the prevalence, incidence, DALYs, YLDs and YLLs were extracted for different age groups of patients with DN, along with their corresponding 95% uncertainty intervals (UIs). The GBD study plays a crucial role in improving our understanding of the impact of diseases and injuries on population health, and in evaluating progress towards achieving international health goals (12).

All data in this study were obtained from the Global Burden of Disease (GBD) public database. In accordance with the Declaration of Helsinki, since the database has been de-identified and is freely accessible to the public, it has been confirmed that this study is exempt from ethical review procedures and does not require informed consent.

### 2.2 Descriptive analysis

Data on the incidence, prevalence, mortality, DALYs, YLDs, and YLLs of type 2 diabetes-related kidney disease from 1990 to 2021 were extracted from the GBD database. This included the corresponding age-standardized incidence rate (ASIR), age-standardized prevalence rate (ASPR), and age-standardized death rate (ASDR). A stratified analysis by age group and gender was conducted for China from 1990 to 2021 through table analysis.

### 2.3 Joinpoint regression analysis

The joinpoint regression model is a set of linear statistical models used to analyze temporal trends in disease burden caused by DN in people with type 2 diabetes. This model’s computational method uses least squares estimation to determine patterns of change in disease incidence, thereby avoiding the subjectivity inherent in traditional linear trend analysis. By identifying and fitting multiple inflection points in time series data separately, the model can effectively represent trend changes, outperforming single linear regression models. Compared to Poisson regression or time series models that fit a single linear trend, this model better fits the combination of trends in the data series, thereby enabling the assessment of changes in the incidence rate trends of diabetic nephropathy (14). This study used Joinpoint version 5.4.0, which was developed by the Statistical Research and Applications Branch of the National Cancer Institute and is available at https://surveillance.cancer.gov/joinpoint/. The average annual percentage change (AAPC) and annual percentage change (APC) were calculated, along with their respective 95% confidence intervals (CIs). The statistical significance of trends was assessed using AAPC and APC values, alongside their respective p-values. If the p-value was greater than 0.05, the trend was considered statistically insignificant. If the p-value was less than or equal to 0.05, the trend was deemed statistically significant (14).

### 2.4 Age-period-cohort analysis

The APC model is a robust statistical method for assessing trends in birth and death rates across different age groups, periods, and cohorts. Due to the inherent linear relationship between age, period, and cohort, it is not possible to objectively indicate the statistical significance of a particular pattern (15). Researchers have introduced intrinsic estimators, penalty functions, and estimation functions from multiple perspectives to address related issues (16). This study employed the APC model analysis technique, which B. Carstensen explained in detail using a Lexis diagram (17). The period from 1990 to 2021 was divided into five-year intervals, and the total number of cases of incidence or mortality was calculated, as well as the cumulative incidence and death rates for different age groups. The APC model was fitted using the Epi package (version 2.59) in R (version 4.5.0).

### 2.5 Decomposition analysis

This study employed the APC model analysis technique, which B. Carstensen explained in detail using a Lexis diagram (18). The period from 1990 to 2021 was divided into five-year intervals, and the total number of cases of incidence or death was calculated, as well as the cumulative incidence and death rates for different age groups. The APC model was fitted using the Epi package (version 2.59) in R (version 4.5.0). This study analyzed three influencing factors— population age structure, population growth and incidence and death rates—using the incidence and death rates of DN as examples. This method provides a preliminary analysis of the three key factors contributing to the global burden.

### 2.6 Cross-country inequality analysis

The Slope Inequality Index (SII) and Concentration Index (CI) are two standard indicators used to quantify absolute and relative gradient inequality, respectively. They were used to measure the degree of inequality in the distribution of disease burden among countries from 1990 to 2021 (19). The SII quantifies absolute inequality by regressing DALYs (disability-adjusted life years) onto a relative position scale based on the SDI (Human Development Index). The CI measures relative inequality by fitting the cumulative health burden to a Lorenz curve to illustrate the distribution of health outcomes among populations ranked by SDI (20).

### 2.7 Predictive analysis

In order to study future projections of DN burden, we predicted changes in age-standardized incidence and death rates for DN between 2022 and 2036, conducting subgroup analyses by gender. The model was established using the Bayesian Age-Period-Cohort (BAPC) method and analyzed using the BAPC and Integrated Nested Laplace Approximation (INLA) software packages (21). This method outperforms traditional linear models by capturing nonlinear time trends and cohort-specific risk characteristics. It avoids the mixing and convergence issues that are commonly encountered in traditional Bayesian methods using Markov chain Monte Carlo sampling (22).

All analyses and visualizations in this study were performed using R (version 4.5.0) for statistical analysis and Joinpoint software (version 5.4.0). P-values below 0.05 were considered statistically significant.

## 3 Results

### 3.1 Description and analysis of the overall trend of China’s disease burden

In 2021, there were 20,911,520 cases of DN in China (95% UI: 19,184,463–22,605,470), including 354,157 new cases (95% UI: 321,265–382,784) and 107,652 deaths (95% UI: 84,626–134,047). The age-standardized incidence rate (ASIR) for both sexes in China increased from 15.11 to 16.29 per 100,000 people between 1990 and 2021. For males, the increase was from 82 to 90 per 100,000 people over the same period. Meanwhile, the age-standardized prevalence rate (ASPR) decreased by 13% (95% UI: 12–15%) over the same period, falling from 1,214.76 to 1,053.92 cases per 100,000 people. Encouragingly, China’s age-standardized death rate (ASDR) for DN has decreased from 6.83 to 5.64 per 100,000 people between 1990 and 2021, representing a 17% decline in mortality rates. The ASDR decreased significantly among women, from 6.49 to 4.74 per 100,000 people, while it decreased slightly among men, from 7.68 to 7.15 per 100,000 people. These figures suggest that China has made progress in managing CKD. In 2021, China had 2,537,070 DALYs due to DN (95% UI: 2,044,338, 3,072,897). Compared with 1990, age-standardized DALYs decreased by 22% in 2021.During this period, China’s age-standardized YLDs and YLLs also showed a downward trend. **Tables 1** and **2** analyze the trend in the disease burden of DN across all age groups. These tables indicate that the disease burden is higher in males than in females.

**Table 1:** Prevalence, incidence, deaths, YLLs, YLDs and DALYs of DN in China in 1990.

Measure	All-age cases (95% UI)			Age-standardized rates per 100 000 people (95% UI)		
	Total	Male	Female	Total	Male	Female
Prevalence	11890522 (10790476,13078708)	5913926 (5344396,6504759)	5976596 (5412193,6565190)	1214.76 (1109.34,1320.7)	1201.87 (1104.76,1301.29)	1231.54 (1116.32,1340.19)
Incidence	127561 (112718,142654)	66801 (58778,74694)	60759 (53787,68366)	15.11 (13.45,16.8)	16.67 (14.77,18.55)	13.92 (12.4,15.6)
Deaths	43537 (35988,53065)	20352 (15618,26884)	23186 (18631,28722)	6.83 (5.74,8.34)	7.68 (6.05,10.19)	6.49 (5.24,8)
DALYs	1231518 (1022061,1492725)	587754 (450891,751005)	643763 (517496,775929)	155.94 (131.16,188.26)	161.8 (128.23,207.69)	155.3 (125.98,186.62)
YLDs	211844 (145222,274820)	100517 (69407,130209)	111327 (76979,144930)	25.3 (17.55,32.65)	24.6 (17.17,31.88)	25.99 (18.13,33.74)
YLLs	1019674 (825926,1249296)	487237 (365687,636045)	532437 (417476,665185)	130.64 (108.12,158.39)	137.21 (106.85,178.63)	129.31 (102.47,160.19)

**Table 2:** Prevalence, incidence, deaths, YLLs, YLDs and DALYs of DN in China in 2021.

Measure	All-age cases (95% UI)			Age-standardized rates per 100 000 people (95% UI)		
	Total	Male	Female	Total	Male	Female
Prevalence	20911520 (19184463,22605470)	10116315 (9283872,10942400)	10795205 (9866263,11741118)	1053.92 (971.11,1139.64)	1040.72 (958.25,1124.45)	1069.36 (981.62,1164.77)
Incidence	354157 (321265,382784)	177163 (161643,191530)	176994 (160794,191804)	16.29 (14.92,17.53)	17.12 (15.73,18.47)	15.68 (14.34,16.97)
Deaths	107652 (84626,134047)	56959 (41145,75559)	50693 (37927,64177)	5.64 (4.46,7)	7.15 (5.28,9.36)	4.74 (3.52,6.03)
DALYs	2537070 (2044338,3072897)	1326063 (1025571,1698910)	1211007 (937561,1514550)	122.15 (99.62,146.99)	140.79 (109.38,179.93)	109.35 (84.66,137.07)
YLDs	504046 (349778,664629)	239318 (166497,316760)	264729 (182940,346817)	23.3 (16.29,30.54)	22.99 (16.19,30.34)	23.65 (16.44,30.87)
YLLs	2033023 (1587785,2544174)	1086745 (781801,1463528)	946278 (713035,1199389)	98.85 (78.16,123.45)	117.81 (84.66,156.1)	85.7 (64.53,109.07)

**Figure 1:**
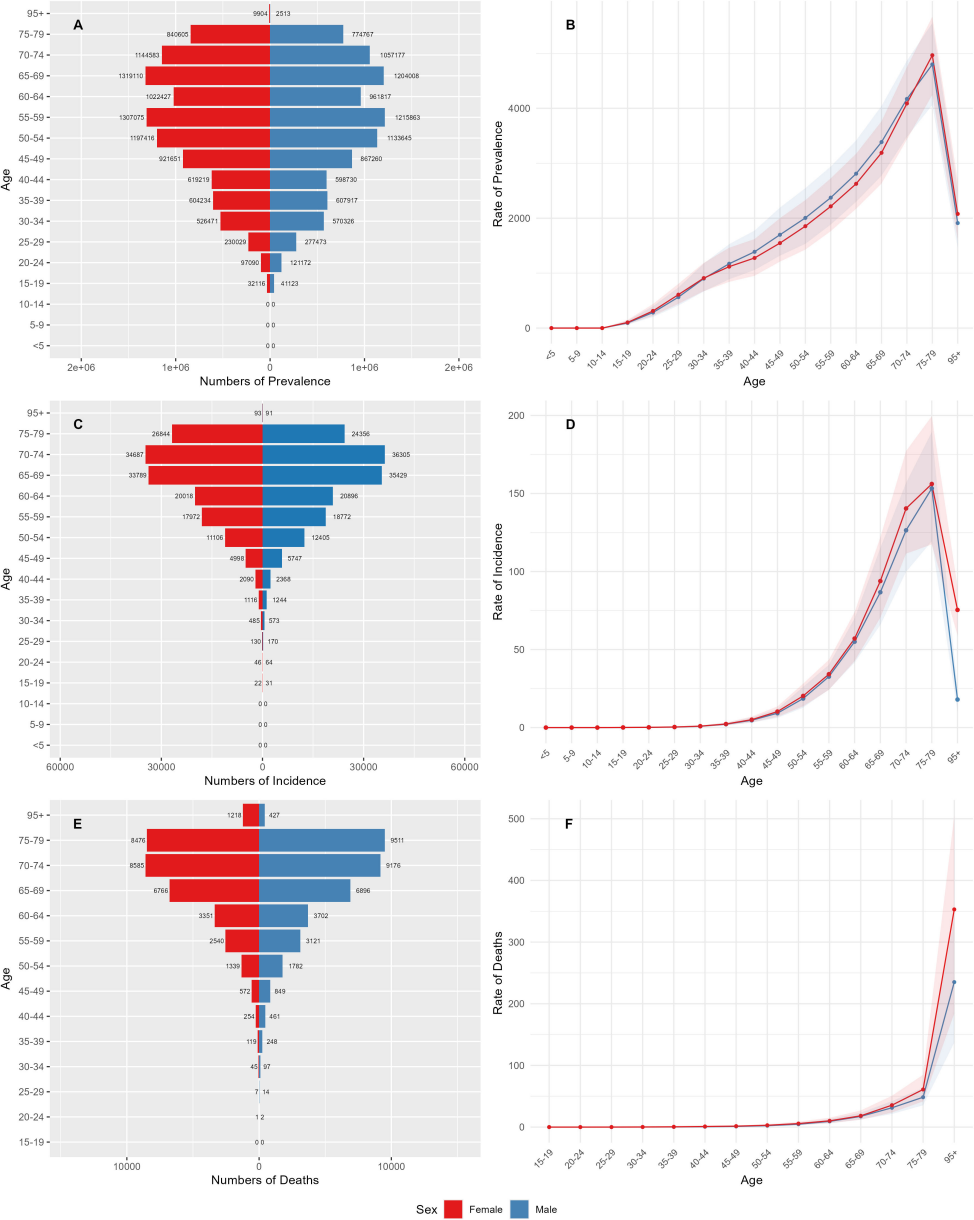
Age-specific incidence, prevalence and death rates for DN in China. **(A)** Age-specific incidence. **(B)** Age-standardized incidence. **(C)** Age-specific prevalence. **(D)** Age-standardized prevalence. **(E)** Age-specific deaths. **(F)** Age-standardized deaths.

**Figure 1** shows the prevalence, incidence and death rates of DN among different age groups of Chinese men and women in 1990 and 2021. The highest number of cases was observed in individuals aged 55 and over. The highest prevalence was found among women in the 65–69 age group and among men in the 55–59 age group (**Figure 1A**). The incidence rate was higher for both men and women in the 70–74 age group (**Figure 1C**). Mortality rates increased significantly in individuals aged 70 and over (**Figure 1E**). From 1990 to 2021, the ASPR and the ASIR were highest in the 75–79 age group. Both the ASPR and the ASIR increased with age up to the age of 70 (**Figures 1B, D**). From age 35 onwards, the ASPR for men was significantly higher than for women, but after age 70, the ASPR for women surpassed that for men (**Figures 1B, D**). From age 79 onwards, the ASDR showed a significant increase, with women surpassing men (**Figure 1F**).

**Figure 2:**
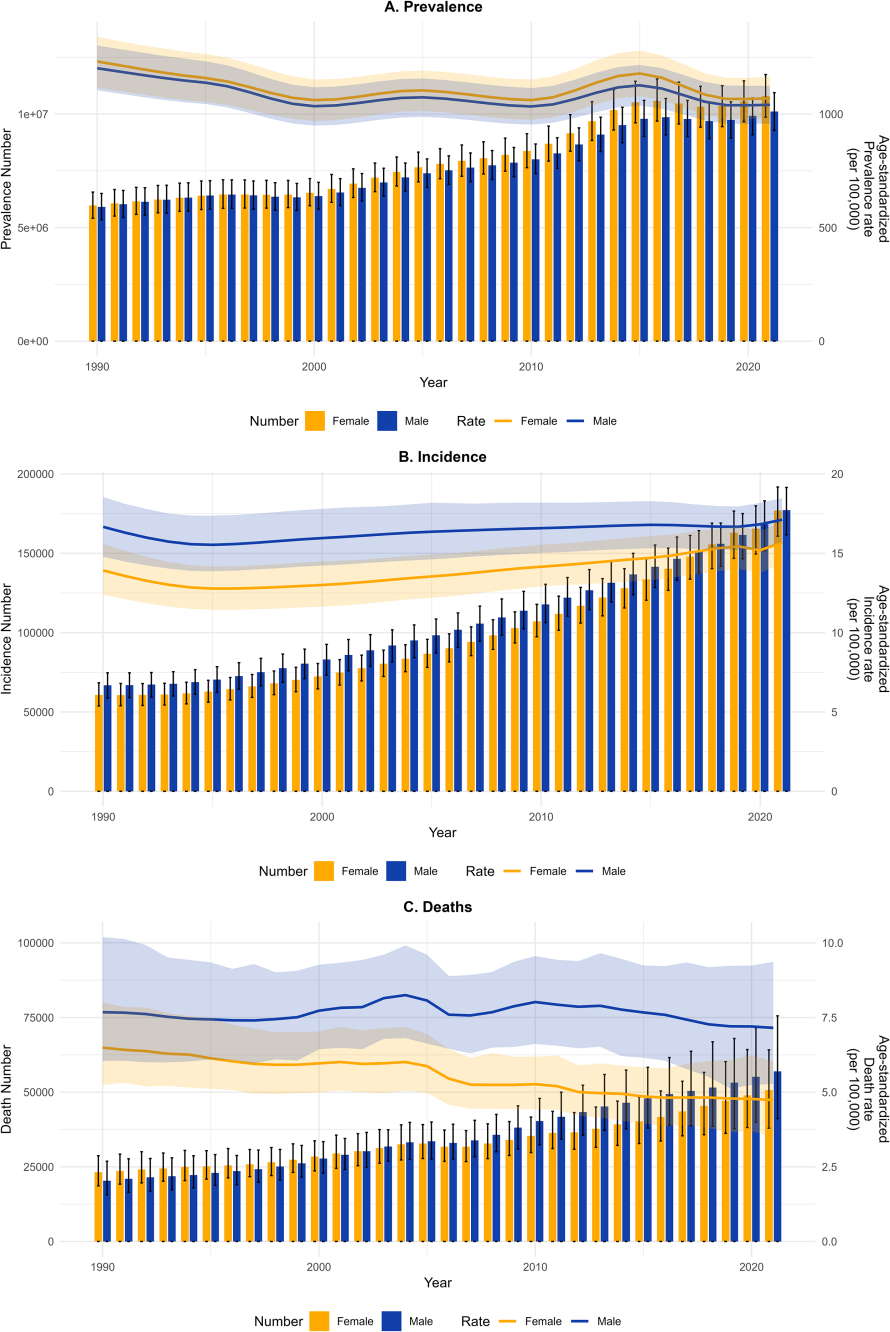
Trends in the prevalence, incidence, deaths and DALYs of diabetic nephropathy in China from 1990 to 2021, categorized by gender and age group. **(A)** Number of cases and prevalence. **(B)** Number of new cases and incidence. **(C)** Number of cases and death.

**Figure 2** shows trends in the incidence, prevalence, deaths and disease burden of DN among Chinese men and women of all ages from 1990 to 2021. From 1990 to 2021, the number of cases and deaths in all age groups of men and women in China increased continuously (**Figure 2**). In terms of prevalence, women showed higher ASPR than men, and ASPR for both sexes fluctuated dynamically, but there was no significant difference between the two (**Figure 2A**). After 1994, the prevalence of DN showed a mild, sustained upward trend, with men consistently exhibiting higher ASIR than women (**Figure 2B**). There were significant gender differences in death rates, with men having higher ASDR than women. Male ASDR increased significantly between 1990 and 2004, but has declined since 2010. The trend in women was similar to that in men (**Figure 2C**).

### 3.2 Joinpoint regression analysis

**Figure 3:**
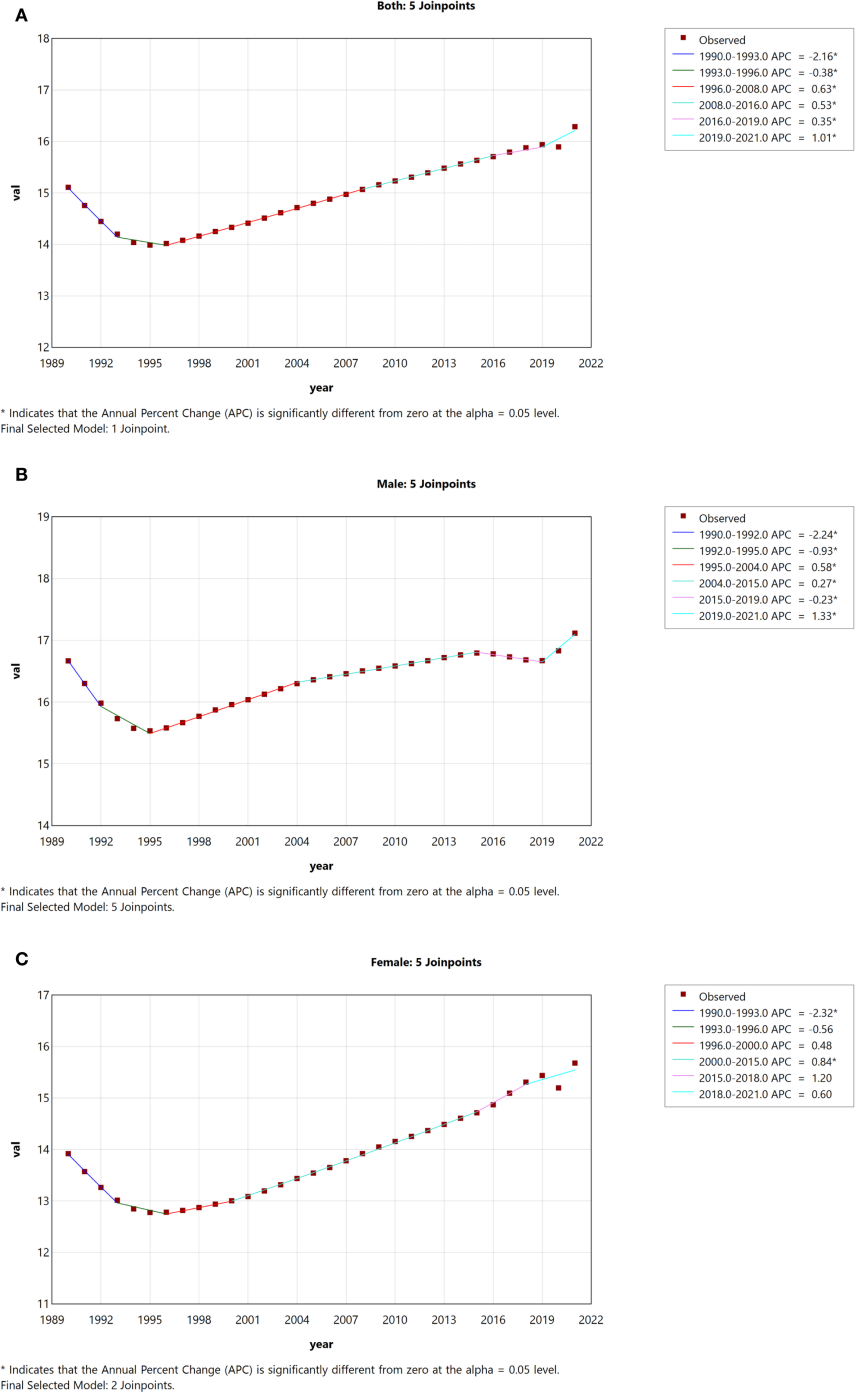
Joinpoint regression analysis of gender-specific age-standardized incidence rates of DN in China from 1990 to 2021. **(A)** Age-standardized incidence rates for both sexes. **(B)** Age-standardized incidence rates for males. **(C)** Age-standardized incidence rates for females.

**Figure 4:**
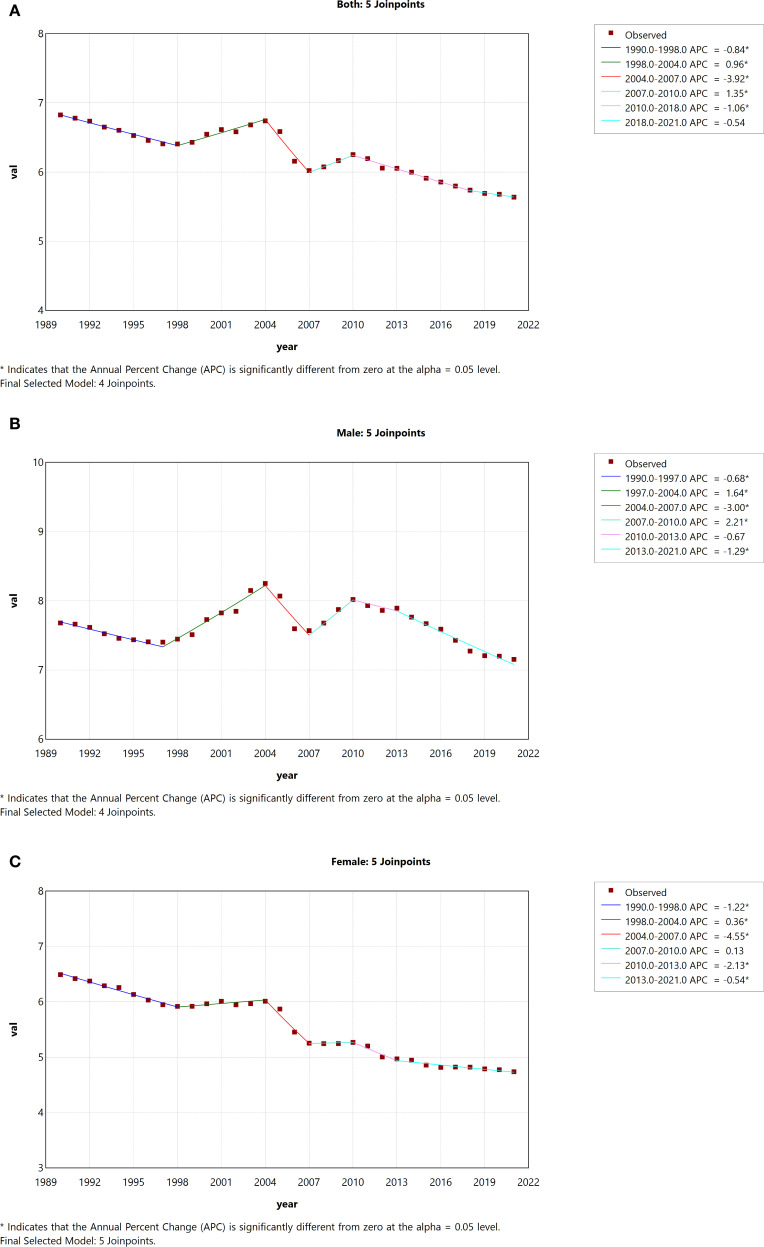
Joinpoint regression analysis of gender-specific age-standardized death rates for DN in China from 1990 to 2021. **(A)** Age-standardized death rates for both sexes. **(B)** Age-standardized death rates for males. **(C)** Age-standardized death rates for females.

**Tables 2** and **3** show trends in the incidence and mortality rates of DN in China from 1990 to 2021, as well as analyses of the annual percentage changes (APCs).In China, we observed a significant decline in ASIR among both men and women from 1990 to 1996 (1990-1993:APC = -2.16; 1993-1996: APC = -0.38), followed by a significant increase from 1996 to 2021 (1996-2008: APC = 0.63; 2008-2016: APC = 0.53; 2016-2019: APC = 0.35; 2016-2021: APC = 1.01) (**Figure 3A**). ASIRs for both males and females showed a significant increase (male AAPC = 0.08, 95% CI: 0.06, 0.10; female AAPC = 0.36, 95% CI: 0.09, 0.63; p < 0.05) (**Figures 3B, C**). From 1990 to 2021, the ASDR for both males and females showed an overall downward trend (AAPC = −0.61, 95% CI: −0.80, −0.43) (**Figure 4A**). The ASDR for females decreased significantly between 2004 and 2007 (APC = −4.55), after which there was a slight increase in death rates between 2007 and 2010 (APC = 0.13). Thereafter, the ASDR continued to decline (**Figure 4C**). Similarly, the ASDR in males showed a significant decline from 2004 to 2007 (APC = -3.00), followed by a significant increase from 2007 to 2010 (APC = 2.21). Subsequently, the decline was more pronounced compared to females (**Figure 4B**). While the ASDR in females showed a significant decrease (AAPC = -1.03, 95% CI: -1.27, -0.79), the ASDR in males fluctuated considerably without a clear trend (AAPC = -0.27, 95% CI: -0.55, 0.01; p > 0.05) (**Figures 4B, C**).

**Table 3:** Joinpoint regression analysis: Trends in ASIR and ASDR (per 100,000 people) among Chinese men and women, 1990–2021.

Gender	ASIR			ASDR		
	Period	APC (95% CI)	AAPC (95% CI)	Period	APC (95% CI)	AAPC (95% CI)
Both	1990-1993	-2.16(-2.25,-2.06)*	0.23(0.20,0.26)*	1990-1998	-0.84(-0.99,-0.69)*	-0.61(-0.80,-0.43)*
	1993-1996	-0.38(-0.57,-0.19)*		1998-2004	0.96(0.69,1.24)*	
	1996-2008	0.63(0.62,0.64)*		2004-2007	-3.92(-5.12,-2.70)*	
	2008-2016	0.53(0.51,0.55)*		2007-2010	1.35(0.10,2.62)*	
	2016-2019	0.35(0.20,0.50)*		2010-2018	-1.06(-1.23,-0.89)*	
	2019-2021	1.00(0.86,1.14)*		2018-2021	-0.54(-1.39,0.32)	
Male	1990-1992	-2.24(-2.38,-2.10)*	0.08(0.06,0.10)*	1990-1997	-0.68(-0.98,-0.38)*	-0.27(-0.55,0.01)
	1992-1995	-0.93(-1.07,-0.79)*		1997-2004	1.64(1.34,1.95)*	
	1995-2004	0.58(0.56,0.60)*		2004-2007	-3.00(-4.59,-1.36)*	
	2004-2015	0.27(0.26,0.28)*		2007-2010	2.21(0.54,3.92)*	
	2015-2019	-0.23(-0.29,-0.18)*		2010-2013	-0.67(-2.39,1.09)	
	2019-2021	1.33(1.22,1.44)*		2013-2021	-1.29(-1.54,-1.03)*	
Female	1990-1993	-2.31(-3.28,-1.34)*	0.36(0.09,0.63)*	1990-1998	-1.22(-1.40,-1.04)*	-1.03(-1.27,-0.79)*
	1993-1996	-0.56(-2.41,1.33)		1998-2004	0.36(0.02,0.70)*	
	1996-2000	0.48(-0.47,1.45)		2004-2007	-4.55(-5.92,-3.15)*	
	2000-2015	0.84(0.76,0.92)*		2007-2010	0.13(-1.34,1.61)	
	2015-2018	1.20(-0.40,2.82)		2010-2013	-2.13(-3.55,-0.68)*	
	2018-2021	0.60(-0.16,1.36)		2013-2021	-0.54(-0.75,-0.33)*	

AAPC, average annual percentage change over the entire period; APC, annual percentage change; Cl, confidence interval. *Indicates p value < 0.05.

### 3.3 Age, period and cohort analysis of ASIR and ASDR in China

**Figure 5:**
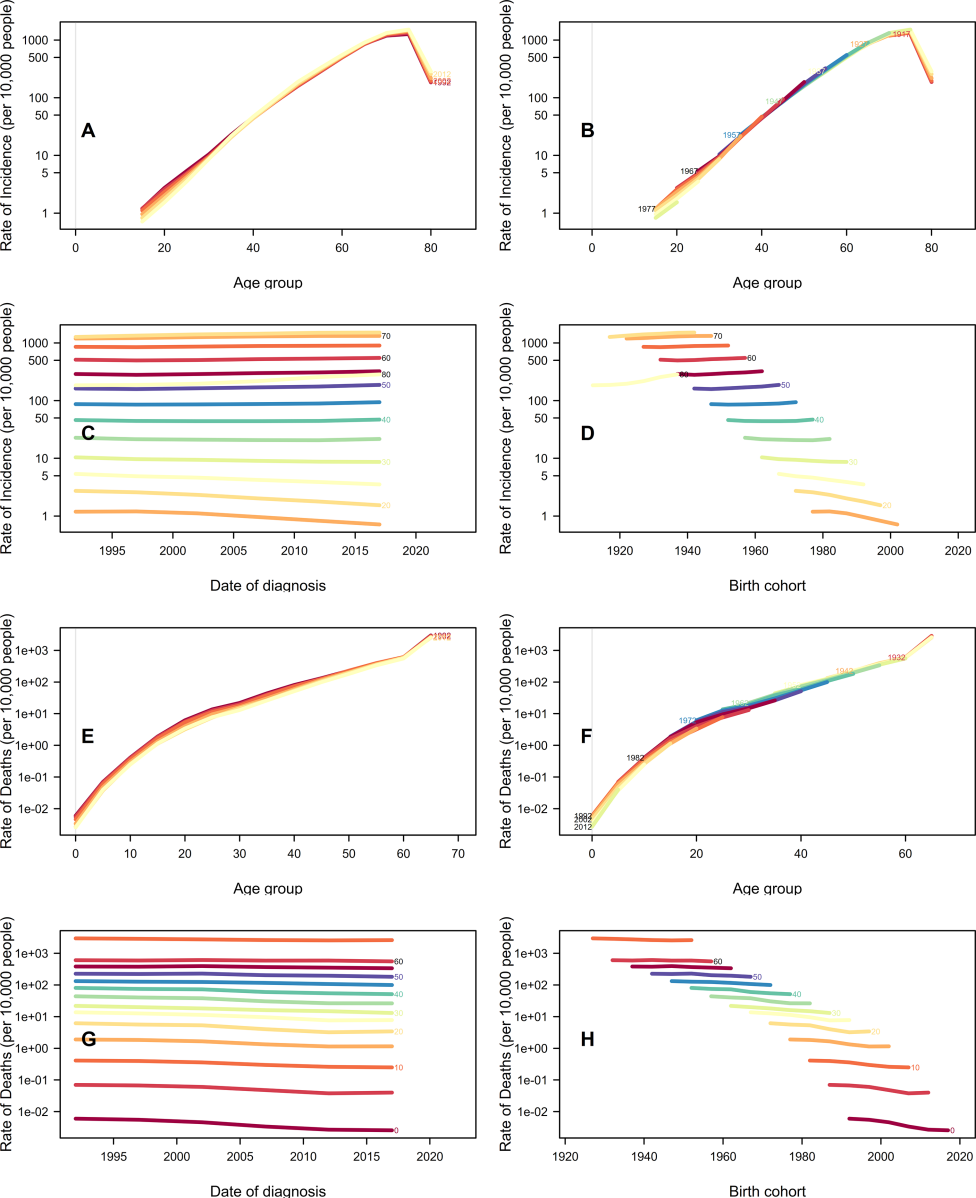
Incidence and death rates of DN in China. **(A)** Age-specific incidence rates by time periods, with each line connecting the age-specific incidence rates for a 5-year period. **(B)** Age-specific incidence rates by birth cohort, with each line connecting the age-specific incidence rates for a 5-year cohort. **(C)** Period-specific incidence rates by age group, with each line connecting the birth cohort-specific incidence rate for a 5-year age group. **(D)** Birth cohort-specific incidence rates by age group; each line connects the birth cohort-specific incidence rate for a 5-year age group. **(E)** Age-specific death rates by time periods; each line connects the age-specific death rate for a 5-year period. **(F)** Age-specific death rates by birth cohort; each line connects the age-specific death rate for a 5-year birth cohort. **(G)** Period-specific death rates by age group; each line connects the birth cohort-specific death rate for a 5-year age group. **(H)** Birth cohort-specific death rates by age group; each line connects the birth cohort-specific death rate for a 5-year age group.

**Figures 5A** and **E** illustrate the incidence and death trends of DN across various age groups from 1992 to 2017. It is worth noting that the incidence rate initially increases and then decreases, whereas the death rate increases sharply with age. **Figures 5B** and **F** clearly show the incidence and death rates of DN in different age groups, highlighting the differences between them. **Figures 5C, G** illustrate the long-term trends in DN incidence and death between 1990 and 2021. In China, the highest incidence of DN is in the over-70s age group, and the incidence rate has remained stable over time. Meanwhile, the death rate in the under-40 age group shows a slight downward trend, while the death rate in the over-50 age group remains stable. **Figures 5D, H** show the incidence and death rates over time for different age groups. In younger age groups, the incidence rate decreases as the birth year increases, whereas in older age groups, it remains relatively stable. However, the death rate decreases across all age groups.

### 3.4 Decomposition analysis of China

**Figure 6:**
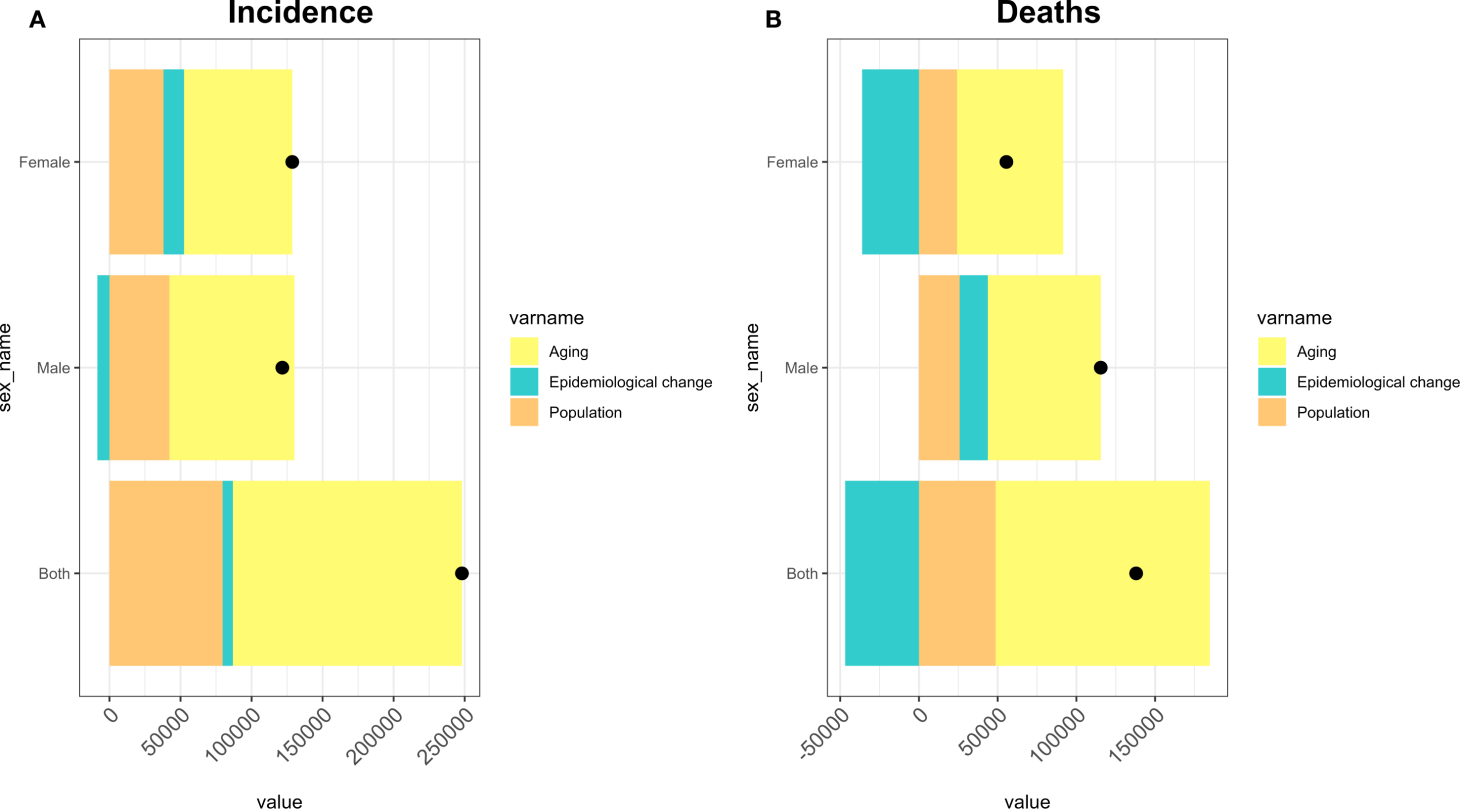
Decomposition analysis of Chinese DN by gender from 1990 to 2021. **(A)** Incidence rate in China. **(B)** Death rate in China. Black dots represent the total change in population growth, ageing, and epidemiological changes.

This study uses a decomposition analysis method to break down changes in DN incidence and mortality rates into contributions from population growth, population ageing, and epidemiological changes (**Figure 6**). In China, population ageing, population growth, and epidemiological changes account for 64.98%, 32.11%, and 2.91% of the changes in incidence rates, respectively, and 98.6%, 35.34%, and -33.94% of the changes in death rates. The analysis indicates that population ageing contributes most significantly to the incidence and death rates of DN in China (64.98% and 98.6%, respectively). In terms of incidence, the proportion of ageing males (72.31%) is significantly higher than that of females (59.16%). In terms of death, ageing is most significant among females (121.41%). Meanwhile, epidemiological changes impacted the overall incidence rate by 2.91% and the death rate by -33.94%. However, the impact on death was more pronounced among women (-64.95%), whereas the overall incidence proportion was 11.23%. Nevertheless, population growth positively impacted both incidence and death rates, with the highest impact observed in female death rates (43.54%) (**Table 4**).

**Table 4:** Decomposition analysis: Changes in the incidence and death of DN in China from 1990 to 2021.

Measure	Sex	Overall_ Difference	Aging (Percent%)	Population (Percent%)	Epidemiological change (Percent%)
Incidence					
	Both	248072.29	161197.5(64.98)	79648.34(32.11)	7226.464(2.91)
	Male	121672.03	87976.55(72.31)	42202.64(34.69)	-8507.16(-6.99)
	Female	128680.22	76128.18(59.16)	38099.88(29.61)	14452.16(11.23)
Deaths					
	Both	137951.7	136020.5(98.6)	48745.48(35.34)	-46814.2(-33.94)
	Male	115487.5	71709.22(62.09)	25823.04(22.36)	17955.26(15.55)
	Female	55547.31	67442.24(121.41)	24185.78(43.54)	-36080.7(-64.95)

### 3.5 Cross-national inequality analysis

**Figure 7:**
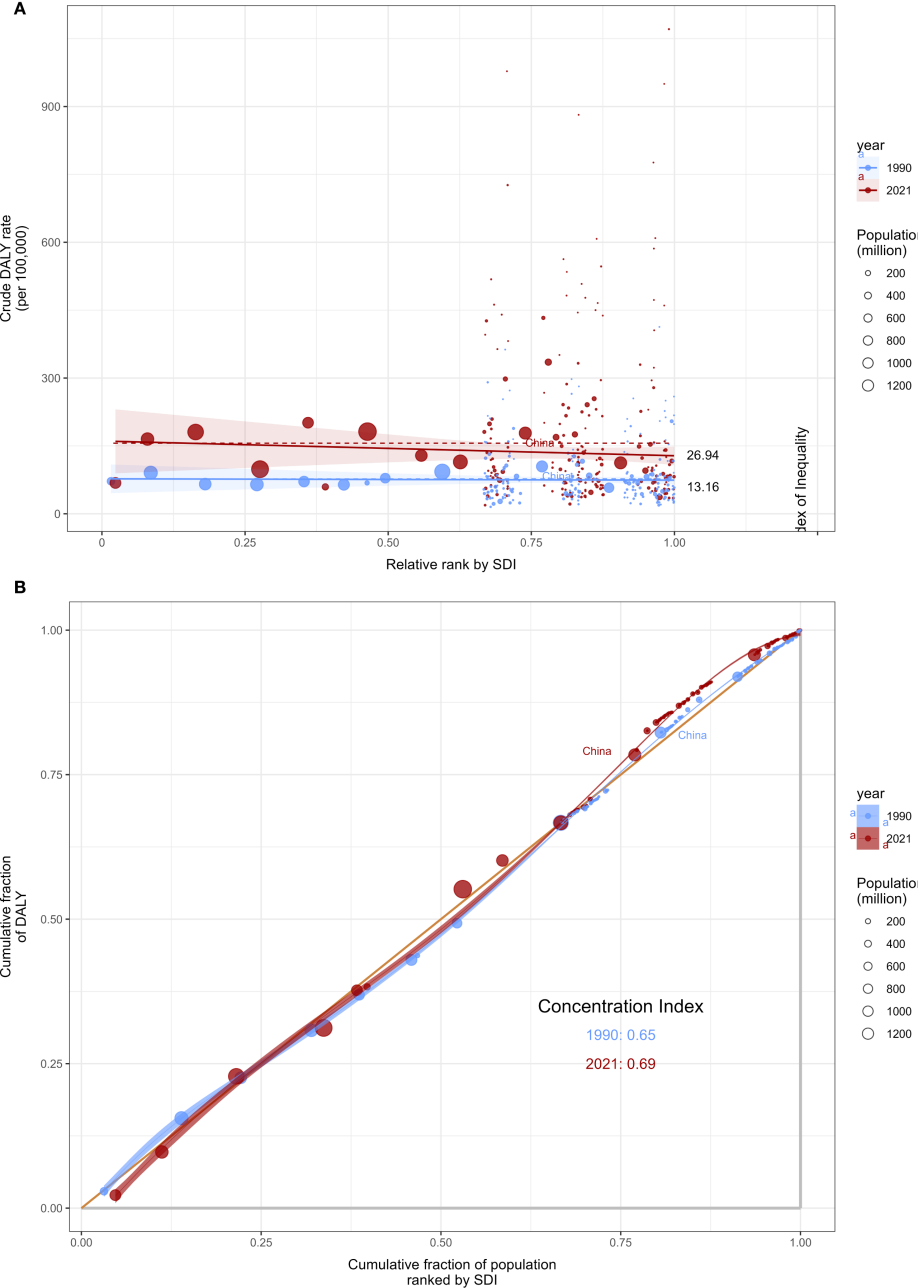
Regression curve **(A)** and concentration index plot **(B)** of health inequalities in disability-adjusted life years (DALYs) for DN worldwide from 1990 to 2021.

Analysis shows that there is significant differentiation in the burden of DN disease between countries with different SDI levels. This inequality based on socio-economic development is showing a continuing trend of deepening, both in absolute and relative terms. The Social Inequality Index (SII) indicated that in 1990, countries with higher SDI levels had lower DALYs for DN, but by 2021, countries with higher SDI levels exhibited higher DALYs. In 1990, the SII was 13.16 cases per 100,000 people, but by 2021, the SII had increased to 26.94 cases per 100,000 people, indicating that the gap between countries with different SDI levels had doubled (**Figure 7A**). Meanwhile, the concentration index (CI) increased from 0.65 in 1990 to 0.69 in 2021 (**Figure 7B**). The above results suggest that absolute and relative health inequalities in DN are increasing and still exist, indicating that health inequalities between different regions are gradually widening. It will pose significant challenges for the future diagnosis and treatment of DN. In the process of globalization, economic development has not alleviated health inequalities, but has instead exacerbated disease burden disparities. The shift in disease patterns in populous countries such as China further amplifies this trend, prompting the need for SDI-stratified intervention strategies to achieve health equity.

### 3.6 Predictive analytics

**Figure 8:**
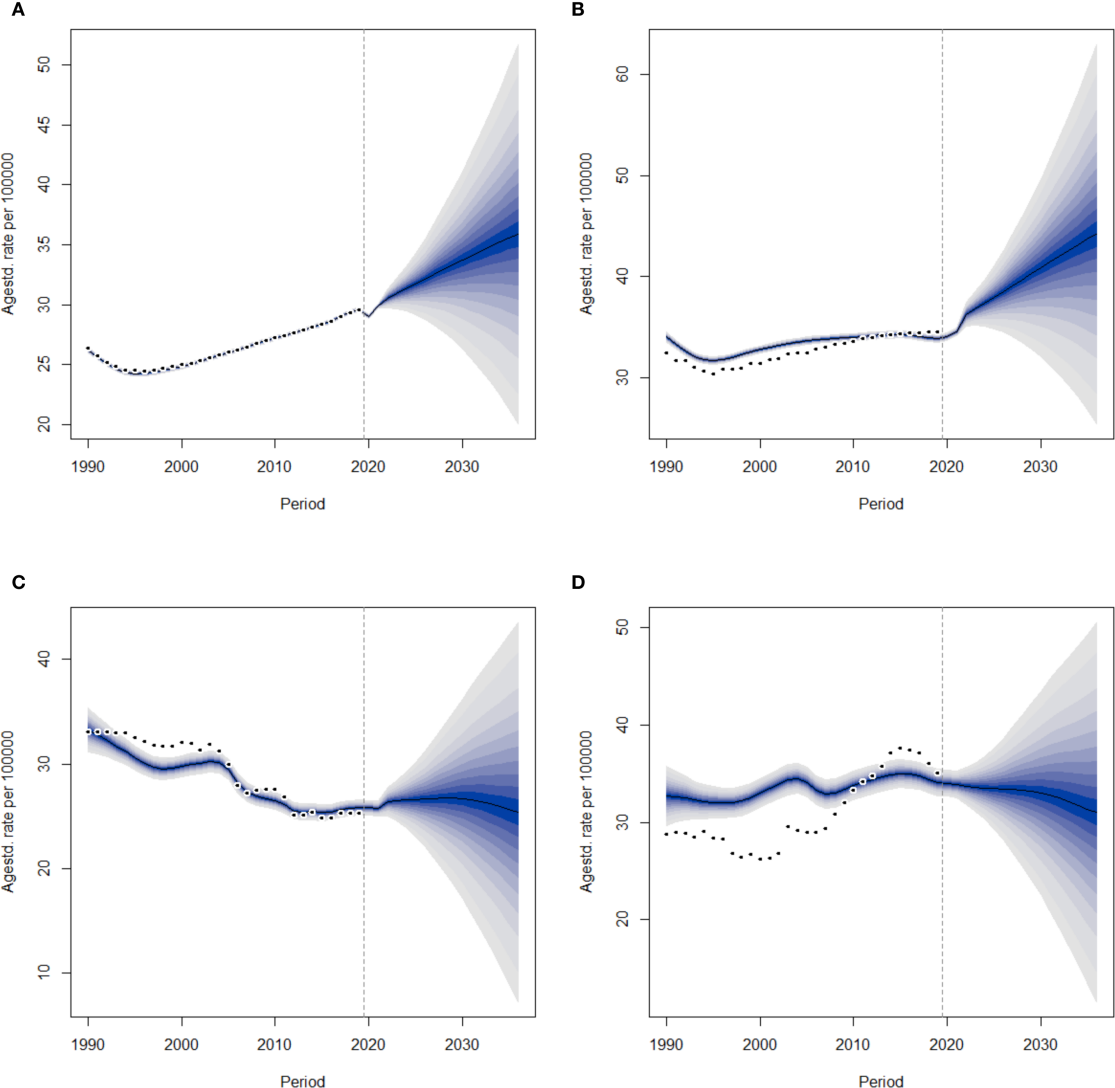
BAPC model predictions for the incidence and death rates of DN in Chinese men and women from 2022 to 2036. The shaded areas around the lines indicate the 95% confidence intervals. **(A)** Female ASIR. **(B)** Male ASIR. **(C)** Female ASDR. **(D)** Male ASDR.

This study applied the Bayesian age-period-cohort (BAPC) model to systematically predict the ASIR and ASDR of DN for different gender groups in China from 2022 to 2036, with the aim of revealing the future direction of the disease burden. According to the prediction model, the ASIR of DN is expected to show a sustained upward trend in the future. The incidence rate among the female population is projected to increase annually, with the trend becoming more pronounced over time (**Figure 8A**), suggesting that the incidence rate of DN among women may continue to rise. The trend for males is even more concerning, with a significant increase in incidence expected around 2036 (**Figure 8B**). Throughout the entire forecast period, the incidence rate among males was significantly higher than that among females, indicating that the burden of DN among males will be heavier in the future. Overall, the ASDR for DN showed a downward trend, but the ASDR for males was still expected to be higher than that for females (**Figures 8C, D**). Although mortality rates are expected to decline in the future, the future disease burden of DN remains severe. By 2036, the incidence rate of new DN cases is expected to continue to rise, indicating the need for sustained and effective prevention and management strategies.

## 4 Discussion

This study used the GBD 2021 database to systematically assess the trends in the burden of DN in China from 1990 to 2021, and to predict the development trends from 2022 to 2036. The study results show that the disease burden of DN in China continued to rise in 2021. It is consistent with previous findings that the ASIR and ASPR of this disease are increasing in China and across the globe (23). These findings are consistent with those of the IDF report. The increasing burden of DN is due to several factors, including population ageing, dietary habits, accelerated urbanization, lack of physical activity, obesity and the rapid increase in diabetes prevalence (24, 25). In 2021, the total number of cases of DN in China reached 20,911,520, nearly double the figure from 1990. The ASIR for both sexes has increased significantly, which may be attributed to advancements in modern diagnostic technologies and continuous improvements in public health awareness (26). However, the ASPR, ASDR and DALYs have decreased since 1990. It may be due to early detection and intervention using low-cost methods, as awareness of the disease burden and associated risk factors has increased among the relevant population, helping to avoid or at least delay the adverse health consequences of kidney failure (27). Early intervention and treatment can effectively delay the onset and progression of DN and improve quality of life. This study also found that the burden on men was significantly higher than that on women, which may be related to male androgens and unhealthy lifestyle factors such as smoking and drinking (25). Therefore, there are greater challenges in the diagnosis and treatment of DN in Chinese men, and more attention needs to be paid to the disease burden on men.

In addition, our analysis of age distribution shows that the incidence of DN increases significantly after the age of 15–19. The highest number of cases is found among men aged 55–59 and women aged 65–69. The death rate for DN increases significantly after the age of 79. Not only do elderly patients have a high incidence rate, but they also experience a decline in physical function, resulting in a significantly higher death rate than younger patients. Previous studies have shown that death rates are closely related to age, increasing significantly in older people. With advancing age, the gradual decline in physical function may exacerbate the ageing and the gradual decline in renal function. It could increase the incidence and death of DN (28, 29). Therefore, it is particularly important to develop different intervention and treatment plans for different age groups, adjusting to more proactive treatment methods to alleviate the burden of DN in China.

Joinpoint regression analysis shows that the incidence of DN in China has steadily increased between 1990 and 2021. The key turning point was 1995. Prior to this, the incidence of DN declined year on year, but after 1995, there was a significant increase. Subgroup analysis showed that the annual incidence rate tended to increase in both men and women. Some studies have suggested that this may reflect higher health awareness and access to medical resources, which has led to an increase in detection rates (30). The death rate for DN in China fluctuated significantly between 1990 and 2021. A key turning point occurred in 2004. Following this year, the death rate gradually decreased. Subgroup analyses indicated that the death rate among men decreased more slowly than that among women. Studies suggest that this is largely related to the development of China’s new rural cooperative medical system (NRCMS) starting in 2003, which is part of the rural medical insurance system. The national medical insurance coverage rate was 29.7% in 2003, increased to 95.7% by 2011, and has remained above 95% since 2013 (31, 32). These health management and prevention measures can effectively delay the progression of DN and help to manage the condition more effectively.

We also analyzed the impact of population growth, ageing, and changes in epidemiological trends on the burden of DN in China from 1990 to 2021. The results of the decomposition analysis in China show that population ageing is the main contributor to the incidence of DN in China (64.98%), followed by population growth (32.11%). By contrast, epidemiological changes are the main contributor to the mortality rate of DN in women (121.41%). Previous studies have shown that China is facing serious medical challenges due to its ageing population. By 2050, the number of Chinese citizens aged 65 and over will reach 40 million, of which 15 million will be over 80 years old (33). At the same time, population growth has also put pressure on the healthcare system. Although the impact of epidemiological changes is relatively low, rapid population growth may still lead to a significant increase in the burden of DN (34). Population ageing is a major driving factor, and long-term comprehensive strategies need to be formulated based on national circumstances.

Nowadays, the global burden of DN continues to increase. Analysis of cross-national inequalities shows that, although the health inequalities associated with DN have improved compared to previous years, differences in the burden of DN still exist between countries at different stages of social and economic development (SDI). This indicates that the burden of DN is in a state of dynamic fluctuation across countries at different stages of socioeconomic development. Studies have shown that socioeconomic status has a significant impact on the burden of diabetes and its related complications, particularly in countries with a low SDI, which experience a greater burden of DN (35). Socioeconomic status remains an important factor in the burden of DN. The global burden of DN remains severe in China, prompting us to adopt effective treatment strategies early on and ensure early diagnosis and treatment to slow its progression.

Predicting future trends in DN is crucial for effective prevention and treatment strategies. According to a predictive analysis using the BAPC model, the ASIR of DN in China is expected to rise between 2021 and 2036, with a higher incidence rate among men than women. However, the ASDR is projected to decrease, with a slower decline among men. This suggests that men may bear a greater disease burden in the future. It aligns with previous studies indicating that China’s mortality rate has decreased rather than continued to worsen compared to other developing countries (25). It is attributed to gender differences in health behaviours and the influence of sex hormones: oestrogen may protect blood vessels, while testosterone may have adverse effects (36). Furthermore, women exhibit higher nitric oxide (NO) availability and lower oxidative stress levels, potentially mitigating the progression of DN (37). China has clearly achieved good results in the treatment and management of DN. Therefore, China must continue to strengthen science popularization, raise health awareness and encourage healthy lifestyles among its citizens, and continue to improve the health plans of the medical and health system and formulate effective treatment plans. These improvements will effectively slow down the occurrence and development of DN, thereby reducing the disease burden in China.

## 5 Limitations

We used the GBD 2021 database to conduct a comprehensive assessment of the burden of DN in China and to predict incidence and mortality rates over the next 15 years. However, our study also has some limitations. Firstly, it should be noted that the quality of GBD data is affected by inconsistencies in the methods used to collect raw data in different countries, which may lead to discrepancies between the final assessment results and the actual disease burden. Secondly, as a populous country with a diverse ethnic makeup and varied lifestyles, China lacks sufficient data in the GBD 2021 database, particularly at the provincial and municipal levels, making it impossible to analyze differences in DN between urban and rural areas in China. Thirdly, the increase in early screening rates for DN in recent years has led to the prediction results being affected by many uncontrollable factors. Fourthly, as the GBD database is constantly updated, long-term monitoring and management are required.

## 6 Conclusion

This study systematically assessed the evolution of the disease burden of DN in China from 1990 to 2021 and predicted future trends over the next 15 years. The data showed that although the ASDR decreased, indicating progress in prevention and control measures, the incidence rate continued to rise, and the disease burden was significantly higher in male patients than in female patients. Population ageing is the primary factor contributing to this overall increase in the burden of DN in China. Although early diagnosis and treatment have alleviated some of the burden, China’s large population and severe ageing mean that these findings warn of DN becoming a major challenge for the country’s public health system. To achieve health equity goals and reduce the disease burden of DN in China, it will be necessary to adopt a tiered prevention and control strategy in the future, focusing on high-risk groups and areas with weak medical resources.

## Data Availability

The original contributions presented in the study are included in the article/supplementary material. Further inquiries can be directed to the corresponding author.
